# Does a Very Short Length of Abstinence Improve Assisted Reproductive Technique Outcomes in Infertile Patients with Severe Oligo-Asthenozoospermia?

**DOI:** 10.3390/jcm10194399

**Published:** 2021-09-26

**Authors:** Federica Barbagallo, Aldo E. Calogero, Rosita A. Condorelli, Ashraf Farrag, Emmanuele A. Jannini, Sandro La Vignera, Claudio Manna

**Affiliations:** 1 Department of Clinical and Experimental Medicine, University of Catania, 95123 Catania, Italy; federica.barbagallo11@gmail.com (F.B.); acaloger@unict.it (A.E.C.); rosita.condorelli@unict.it (R.A.C.); sandrolavignera@unict.it (S.L.V.); 2 Biofertility IVF and Infertility Center, 00198 Rome, Italy; ashraf_farrag@yahoo.com; 3 Department of Systems Medicine, University of Rome “Tor Vergata”, 00133 Rome, Italy; eajannini@gmail.com; 4 Department of Biomedicine and Prevention, University of Rome “Tor Vergata”, 00133 Rome, Italy

**Keywords:** ART, sperm parameters, ICSI, fertilization rate, pregnancy rate

## Abstract

In recent years, a growing number of studies seem to support the beneficial effects of a very short abstinence period on sperm parameters, especially in patients with oligo-asthenozoospermia (OA). On this basis, the aim of this study was to evaluate the effects of a short period of abstinence (1 h) on intracytoplasmic sperm injection (ICSI) outcomes in infertile patients with severe OA. We performed a retrospective study on 313 ICSI cycles in which couples were divided into two different groups based on sperm parameters of the male partners. Group 1 included normozoospermic men or male partners with a mild OA (*n* = 223). Group 2 included male partners with severe OA (*n* = 90). They were asked to provide a second consecutive ejaculation after 1 h from the first one. The best ejaculate was used to perform ICSI. We found a significant increase of total (*p* < 0.001) and progressive motility (*p* < 0.001) in the second ejaculate of patients of Group 2 compared with those of the first one. Spermatozoa of the second ejaculate were chosen for ICSI for all patients in Group 2. We found statistically significant improvement of clinical pregnancy rate (*p* = 0.001) and embryo quality (*p* = 0.003) in couples in Group 2 compared to those of Group 1. No statistically significant difference was found in fertilization, implantation, live birth delivery, and miscarriage rates between the two groups. Therefore, a second semen sample collected after a very short time-interval in patients with severe OA allowed us to obtain significantly higher clinical pregnancy rate with improved embryo quality compared to normozoospermic men or patients with mild OA. Fertilization, implantation, live birth delivery, and miscarriage rates were similar between the two groups. The present study shows that a second consecutive ejaculate could represent a simple strategy to obtain better sperm parameters and assisted reproductive technology (ART) outcomes in infertile patients with mild-severe OA.

## 1. Introduction

In the second decade of the new millennium, infertility remains a global public health issue, affecting approximately 15% of all couples in the reproductive age in industrialized countries [1]. In recent decades, assisted reproductive technologies (ART) have spread worldwide to help infertile couples to achieve pregnancy [2]. A proper diagnostic and therapeutic workup associated with adequate preparation of infertile couples before ART is essential to obtain spermatozoa and oocytes of high quality and, in turn, to improve the outcomes of ART. The female factor has often been considered as the main factor responsible for the failure of ART cycles. However, a male factor is responsible for couple infertility in about half of the cases [1]. A breakthrough in this regard was the development of intracytoplasmic sperm injection (ICSI) for infertile patients with severe oligoasthenoteratozoospermia (OAT) [3]. A better sperm quality enhances fertilization rate and embryo quality following ICSI [4]. In fact, impairment of sperm parameters may affect fertilization and cleavage rates and the quality of embryos [5]. Furthermore, it can be associated with higher aneuploidy [6] and miscarriage rates [7]. In clinical practice, several antioxidants are commonly used to improve male fertility before ART. Oral antioxidant treatment appears to improve ICSI outcomes, especially in patients with sperm DNA damage, in whom antioxidants reduce the percentage of damaged spermatozoa [8]. A position statement of the Italian Society of Andrology and Sexual Medicine placed a high value on the administration of antioxidants in patients with idiopathic infertility in the presence of documented abnormal sperm parameters and altered sperm DNA fragmentation after an adequate diagnostic procedure [9]. Therefore, a correct diagnostic and therapeutic management of infertile patients before ART is essential to increase success rates, without simply leaving ICSI to solve the severe male factor of infertility. 

Due to the increasing number of infertile couples undergoing ART, several studies have focused on identifying factors that can improve the outcome of these techniques. Among the different factors that affect sperm quality, sexual abstinence is often overlooked, although the length of sexual abstinence has been shown to influence sperm parameters. The World Health Organization (WHO) recommends that semen should be collected for its analysis after a minimum of 2 days and a maximum of 7 days of sexual abstinence [10]; however, the European Society of Human Reproduction and Embryology (ESHRE) advises an abstinence period of 3–4 days only [11]. The basis for these recommendations is unclear and the current indications on the abstinence length should be revisited [12]. Many years ago, McLeod and Gold indicated that the abstinence period should be based on copulation frequency [13]. They reported that a coital frequency less than three times per week may result in delayed fertility by missing the ovulatory window and/or by impairing sperm parameters [13]. 

Many studies have investigated the influence of the length of sexual abstinence on sperm parameters, although the results are controversial. In fact, a longer abstinence period seems to improve semen fluid volume and sperm count whereas the effects on motility, morphology, and DNA fragmentation are contradictory [14]. However, these studies are extremely variable both for the characteristics of men enrolled and for the length of sexual abstinence [15]. 

A growing number of studies are focusing their attention on the possibility of using a second ejaculate collected after a very short period of abstinence in infertile men, especially patients with oligo-asthenozoospermia (OA). We previously reported that a second consecutive ejaculate (collected within 1 h from the first) resulted in better conventional sperm parameters (motility and morphology) and a lower percentage of spermatozoa with fragmented DNA in normozoospermic male partners of infertile couples and even more in patients with OAT [16]. Our findings were in line with the most recent literature [4,17,18,19,20,21,22,23,24,25]. 

However, the optimal length of abstinence for couples undergoing ART cycles is still debated. Short abstinence in terms of days [26,27] or hours [28] has improved the pregnancy rate by intrauterine insemination. A recent meta-analysis described that a short abstinence period (less than 4 days) was associated with higher implantation (*p* = 0.0001) and pregnancy rates (*p* = 0.0006) compared to a period of abstinence of 4–7 days in ART treatments [29]. 

Few studies have evaluated the effect of a very short period of abstinence on ART outcomes. Sugyam et al. reported that the fertilization rate using spermatozoa collected after 30–60 min (53.3%) was significantly higher than that from the first (28.9%, *p* < 0.05) [4]. A very recent study evaluated the effect of an abstinence period of 2 h on sperm parameters and ART outcome in patients with severe OAT [25]. Their findings suggest that a very short abstinence period in severe OAT allows the obtaining spermatozoa with better quality, especially in terms of motility and, in turn, achievement of the same probability of pregnancy compared to normozoospermic patients [25]. 

Moreover, an increase of the euploid blastocysts has been reported using ejaculates obtained after very short abstinence (1 h) [15]. 

On this basis, this study aimed to evaluate the effects of a very short period of abstinence (1 h) on intracytoplasmic sperm injection (ICSI) outcomes in infertile patients with severe OA.

## 2. Materials and Methods

### 2.1. Sample Selection

This is a clinical study treating 313 infertile couples with ICSI which we have retrospectively evaluated. All 313 male partners had abstinence of 2–7 days, as suggested by the WHO 2010 criteria. All semen samples were collected by masturbation within the fertility center to minimize conditions that might alter sperm parameters.

All semen analyses were performed by the same experienced embryologist according to the WHO 2010 criteria [10]. The evaluation of sperm motility was performed on a 10 μL drop on a glass slide with a 22 × 22 mm coverslip and heated stage at 37 °C, with a lens with a reticule. The slides were examined with a positive phase-contrast optics at a magnification of 400×. We assessed 400 spermatozoa per replicate for an accurate evaluation of motility. The assessment of morphology was done using Diff Quick stained slides (Medion Diagnostics AG, Dudingen, Switzerland). Morphology was evaluated at a magnification of 400× and 1000×. We prepared multiple slides to obtain at least 400 spermatozoa for a proper sperm morphological evaluation. 

Couples were divided into two groups based on the sperm parameters of the male partners. Group 1 (*n* = 223) included normozoospermic men or patients with mild OA, whereas Group 2 (*n* = 90) included male partners with severe OA. The male partners of Group 2 couples were asked to provide a second consecutive semen sample after explaining to them the possibility of having better sperm parameters in the second ejaculate. Informed consent was obtained from each patient. The second semen collection was obtained 1 h after the first. Then, the second sample was analyzed and compared to the first for the following parameters: concentration (mil/mL), total and progressive motility (%), and morphology (%). 

The ICSI procedure was performed with the best ejaculate and swim-up preparation obtained according to the percentage of spermatozoa with progressive motility.

### 2.2. Controlled Ovarian Hyper Stimulation

Controlled ovarian hyper stimulation protocols were carried out using recombinant follicle-stimulating hormone (FSH, Gonal-F, Merck Serono, Geneva, Switzerland) and gonadotropin-releasing hormone (GnRH) antagonist according to the ovarian reserve. Monitoring of the follicular development was performed by real-time ultrasound scans from day 2 of treatment until the day of follicular aspiration guided by the patient’s response to stimulation. When at least one ovarian follicle reached 18–20 mm diameter, ICSI was performed 36–38 h after the administration of human chorionic gonadotropin (hCG, Gonasi, 10,000 IU IBSA, Lodi, Italy). 

### 2.3. Intracytoplasmic Sperm Injection Procedure

The first semen collection was obtained about 5–6 h before microinjection, scheduled about 40 h after hCG administration. ICSI procedure was performed with spermatozoa obtained by “swim-up” using the first or second ejaculate according to the male partner sperm parameters as above reported. The “swim-up” technique was performed directly from liquefied semen. To accomplish this, several semen aliquots were taken from each sample and placed in tubes underneath an overlay of flushing medium (Origio Italia Srl, Rome, Italy). Round-bottom tubes or four-well dishes were used to optimize the interface surface area between the semen layer and the culture medium. The samples were left to incubate at 37 °C in an incubator for 30–45 min. Spermatozoa with the best motility and ability to migrate were recovered.

The collected cumulus-enclosed oocytes were maintained in 500 μL multi dishes with 4 wells (Nunclon Surface, Roskilde, Denmark) in Continuous Single Culture™ Medium–Complete (CSCM-C) (Irvine Scientific, FujiFilm, Tilburg, The Netherlands) under oil (oil for embryo culture, Fuji Film, Europe) and maintained in the incubator for 2 h after their retrieval. Then they were decumulated in hyaluronidase drops (Hyaluronidase Solution, Fuji Film Europe Europe).

ICSI procedure was performed according to the standard technique. Embryo culture was performed in a standard incubator at 37 °C, 6% CO_2_, 5% O_2_ in CSCM-C (Irvine Scientific, FujiFilm, Tilburg, The Netherlands). Embryo transfer was performed after 2 days of culture. After 36–44 h in culture, all embryos were carefully examined with both a dissecting and an inverted microscope. Embryo grading was carried out according to the system proposed by Puissant and colleagues [30]. The number and size of blastomeres as well as the presence or absence of anucleate fragments, were carefully recorded so that embryos could be scored as follows: 4 = embryos with clear, regular blastomeres and either no fragmentation or a maximum of five small anucleate fragments; 3 = embryos with few or no fragments but with unequal blastomeres (>1/3 difference in size); 2 = embryos with more fragments but over <1/3 of the embryonic surface; 1 = fragments over >1/3 of the embryonic surface. Two points are added if the embryo had reached the 4-cell stage by 48 h after fertilization. 

Then, we compared the ICSI outcomes of couples in which spermatozoa obtained from ejaculate collected after 2–7 days of abstinence (as suggested by 2010 WHO criteria) were used and couples in which a second ejaculate was obtained after a short period of abstinence (1 h). The following primary outcomes were considered: fertilization rate (number of fertilized oocytes/number of oocytes inseminated), implantation rate (number of gestational sacs/number of embryos transferred), clinical pregnancy rate (number of pregnancies with at least one fetal heartbeat/number of pick-up cycles with at least one oocyte retrieved), live birth delivery rate (number of deliveries with at least 1 live birth/number of pick-up with at least 1 oocyte retrieved), miscarriage rate (number of spontaneous abortions/total number of pregnancies), and embryo quality. We also evaluated the type of birth (natural or cesarean) and birth weight. 

### 2.4. Ethical Approval

The study was conducted in the ART Biofertility IVF Center (Rome, Italy) on infertile couples undergoing ICSI treatment. It was reviewed and approved by the Institutional Review Board at the Biofertility IVF Centre who indicated that ethical approval was not required for this study. Data collection followed the principles outlined in the Declaration of Helsinki. All patients provided their informed consent, agreeing to supply their own anonymous information for this and future studies. 

### 2.5. Statistical Analysis

The Kolmogorov–Smirnov test was used to evaluate whether the data were normally distributed. The Student’s *t*-test and the Mann–Whitney U test were used for continuous variables. Dichotomous variables were analyzed by chi-square or Fisher’s exact test. A *p*-value lower than 0.05 was considered statistically significant. The software SPSS 23.0 for Windows (SPSS Inc., Chicago, IL, USA) was used.

## 3. Results

The age of the male partners was statistically significantly different between the two groups (Group 1: 40.5 ± 6.4 vs. Group 2: 38.5 ± 6.7, *p* = 0.017). On the contrary, the age of women in Groups 1 and 2 did not show any statistically significant difference. Likewise, no statistically significant difference was found between antral follicle count (AFC) of women in Groups 1 and 2. Moreover, the total dose of FSH administered to the female partners for the controlled ovarian hyperstimulation was similar between the two groups. Table 1 shows the characteristics of female partners in the two groups. Table 2 shows the sperm parameters in the two groups. Sperm concentration, total and progressive motility were significantly higher in Group 1 than in Group 2. No statistically significant difference was found between the percentage of spermatozoa with normal morphology between the two groups.

The comparison between the first and second semen samples in patients in Group 2 showed a significantly higher percentage of spermatozoa with total (*p* < 0.001) and progressive (*p* < 0.001) motility in the second ejaculate than in the first (Table 3). The percentage of morphologically normal spermatozoa was also higher in the second ejaculate, but the difference did not, albeit only by a small margin, reach statistical significance compared to Group 1 (*p* = 0.054). Therefore, spermatozoa from the second ejaculate were used for ICSI for all 90 patients in Group 2. 

The transfer was performed in all patients enrolled in this study. For patients of Group 1, 586 embryos were transferred (2.7 ± 1.14), whereas 229 embryos (2.6 ± 1.2) were transferred for patients of Group 2. There was no statistically significant difference in the number of embryos transferred between the two groups (*p* = 0.5). 

**Figure 1:**
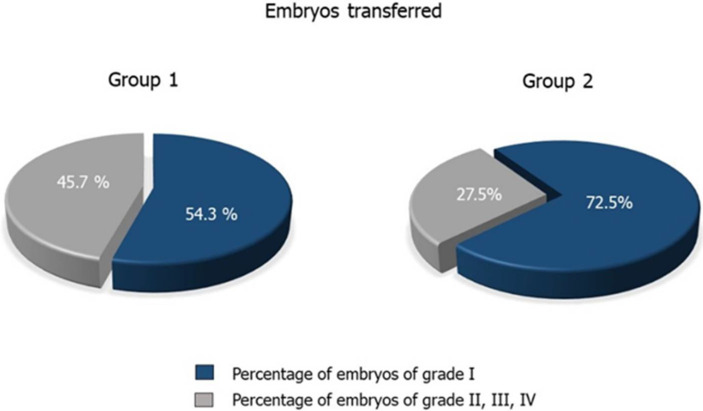
Embryo quality of couples in Groups 1 (*n* = 223) and 2 (*n* = 90).

Fertilization, implantation, clinical pregnancy, live birth delivery, and miscarriage rates are reported in Table 4. The clinical pregnancy rate of couples in Group 2 was significantly higher than that of Group 1 (*p* = 0.001). Interestingly, the former had also a significantly higher percentage of embryos of grade I than couples in Group 1 (*p* = 0.003) (Figure 1). No statistically significant difference was found in fertilization, implantation, live birth delivery, and miscarriage rates between the two groups.

## 4. Discussion

In recent decades, many authors have supported the beneficial effects of a very short abstinence period on sperm parameters. We have previously reported that a second consecutive ejaculate collected within 1 h resulted in an improvement of sperm motility and morphology in normozoospermic male partners of infertile couples. The improvement was more marked in patients with OAT [16]. 

On this basis and according to the recent literature [4,18,19,20,21,22,23,24,25], we conducted this study to evaluate the effects of a very short period of abstinence (1 h) on ICSI outcomes in infertile patients with severe OA. This subset of patients (Group 2) had significant improvement of total (*p* < 0.001) and progressive motility (*p* < 0.001) in the second ejaculate compared with those of the first (Table 3). Based on the increase of sperm progressive motility, we chose to utilize spermatozoa of the second ejaculate in all 90 men of Group 2. These results confirm our previous findings [16]. Most of the studies conducted on OAT patients reported an increase of progressive motility in ejaculate collected after a very short period of abstinence [18,19,20,21,22,23,24,25]; some of these studies also found an increase in normal morphology [20,24,25] and sperm concentration [17,18,21,24,25]. 

Then, we compared ICSI outcomes of couples in Group 1 whose male partners collected their seminal sample after a conventional period of abstinence (2–7 days) and couples in Group 2 whose male partners were requested to provide a second ejaculate collected after 1 h from the first. We found a statistically significant higher clinical pregnancy rate of couples in Group 2 compared to Group 1 (*p* = 0.001), despite the worse seminal parameters of the male partners. No statistically significant difference was found in fertilization, implantation, live birth delivery, and miscarriage rates between the two groups. Thus, a second semen sample collected after 1 h of sexual abstinence allowed the same results in terms of fertilization, implantation, live birth delivery, and miscarriage rates of normozoospermic patients or with mild OA, but increased clinical pregnancy rate, despite their severe OA. A very small number of studies have focused on the effects of a very short period of abstinence on ART outcomes. Barash et al. [31] conducted a study of 39 infertile patients with OAT scheduled for in-vitro fertilization and embryo transfer (IVF-ET). They reported increased fertilization (18.3 ± 25.8 vs. 29.6 ± 29.7; *p* < 0.05), cleavage (0.6 ± 1 vs. 1.9 ± 1.7; *p* < 0.05), and pregnancy (5/14 vs. 0/2; *p* < 0.05) rates when oocytes were incubated with spermatozoa of an ejaculate collected 2 h after the first one, compared with oocytes exposed to spermatozoa from ejaculate collected after 3 days of abstinence [31]. Sugyam et al. reported that the fertilization rate with a second ejaculate collected within 60 min was significantly higher than that from the first (53.3 vs. 28.9%, *p* < 0.05), whereas cleavage rate and embryo quality were comparable [4]. More recently, Ciotti et al. found that using seminal samples collected after an abstinence period of 2 h in severe OAT patients allowed the obtaining of similar rates of fertilization (*p* = 0.59), pregnancy (*p* = 0.34), implantation (*p* = 0.23), and miscarriages (*p* = 0.60) to those achieved in normozoospermic patients [25]. 

Interestingly, we also found a significantly higher percentage of embryos of grade I in Group 2 compared with Group 1 (Figure 1). In a recent study, Scarselli et al. described higher blastocyst euploid formation rates using ejaculates collected after an abstinence length of one hour than by using ejaculates collected after the conventional length of abstinence of 2–5 days (43.6% vs. 27.5%, *p* = 0.043) [15]. The authors hypothesized that this finding may be ascribed to the higher percentage of spermatozoa with mature chromatin in the second ejaculate. Specifically, they used the aniline blue (AB) test to detect protamination and, in turn, chromatin integrity [15]. The chromatin compactness is an important bio-functional sperm parameter for the evaluation of DNA maturity and sperm damage. Staining with AB provides a specific positive reaction for lysine, the main amino acid present in histones, whereas it is little represented in protamines [32]. The histone to protamine transition is a fundamental step in spermatogenesis to facilitate chromatin compaction in the sperm head and, in turn, to prevent the paternal genome from mutagenesis and damage [2]. In fact, DNA complexed with protamine is very stable and resistant to enzymatic digestion. Defects in the histone replacement process may lead to DNA damage and, consequently, to male infertility [2]. Scarselli et al. found a statistically significantly higher percentage of pale-colored spermatozoa (a sign of mature chromatin) in the second ejaculate compared to the first (54.0 ± 1.8% vs. 29.6 ± 2.3%, *p* < 0.005). A lower percentage of spermatozoa with mature chromatin can alter zygote development after ICSI and impair embryo quality [33].

Sperm DNA fragmentation (fDNA) is another bio-functional parameter increasingly used [34] that may be associated with defective maturation, abortive apoptosis, and oxidative stress (OS) [35]. A higher number of spermatozoa with fDNA in couples undergoing ART is associated with worse outcomes [36]. Our previous study reported a decrease of fDNA in both men with normozoospermia and patients with OAT after an abstinence period of 1 h. A lower percentage of spermatozoa with fDNA was reported after recurrent ejaculations every 24 h for four days with final abstinence of 12 h before ICSI [37]. 

Shen et al. found a decreased percentage of spermatozoa with fDNA, an increased total antioxidant capacity (TAC), and higher sperm mitochondrial membrane potential (MMP) in ejaculates from short (1–3 h) compared with long (3–7 days) length of abstinence [23]. MMP is a marker of sperm mitochondrial function that strictly correlates with sperm motility [38].

In agreement with these findings, quantitative proteomic analysis has shown that proteins overexpressed in spermatozoa of the second ejaculate are involved in specific functions, such as sperm motility, capacitation, and antioxidant defense [15]. Human spermatozoa are rich in polyunsaturated fatty acids and, therefore, are very sensitive to damage provoked by reactive oxygen species (ROS). It has been widely shown that an imbalance between oxidative and antioxidant systems in the seminal plasma is detrimental for sperm function and fertility outcome [39]. A short period of abstinence could reduce the time of exposure of spermatozoa to the harmful effects of ROS in the cauda epididymis and, in turn, may result in a “younger” population of spermatozoa [40]. Therefore, the improvement in the sperm quality observed in the second ejaculate could be explained by a different epididymal transit time. Interestingly, Johnson and Varner reported that the sperm transit time through the epididymis was three times longer in patients with oligozoospermia than in men with normozoospermia [41]. Thus, spermatozoa of patients with severe OA stay in the genital tract for a prolonged time and, therefore, suffer more deeply the effects of oxidative stress. This might explain the greater improvement of sperm quality after a very short period of abstinence in patients with severe OA compared with normozoospermic men [16]. During the epididymal transit, sperm maturation includes sperm surface modifications and changes of flagellar beating through which spermatozoa acquire their forward motility and their fertilization capability [42]. In addition, epigenetic modifications occur during the epididymal transit [43] and represent a crucial step during spermatogenesis, sperm maturation, and fertilization process [44]. Shen et al. identified ten kinds of major protein modifications to evaluate the difference in epigenetic modifications in ejaculates after 1–3 h compared to 3–7 days [23]. Notably, sperm butyryl-lysine, propionyl-lysine, and malonyl-lysine modifications were significantly decreased and trimethyl-lysine modifications increased significantly after lower abstinence length [23]. ART failure of couples with male partner infertility could relate to epigenetic modifications found in blastocysts [45]. Epigenetic events in OAT patients directly impact embryogenesis and this could explain the higher miscarriage rates for couples with OAT male partners compared to controls, despite maternal age-match and a similar number of transferred euploid blastocyst [44]. Furthermore, changes in the seminal plasma composition could also have a role in the improvement of sperm motility after a very short period of abstinence. A recent study compared the seminal plasma metabolomics profile in two consecutive ejaculates collected from normozoospermic men after long (4–7 days) followed by a short (2 h) ejaculation-abstinence period. The results showed a lower absolute amount of all metabolites in the second ejaculate. This may be related to the insufficient time available for the secretion and accumulation of these metabolites by accessory sex glands including the epididymis. However, the contemporary lower number of spermatozoa in the second ejaculate resulted in increased absolute amounts of pyruvate and taurine per spermatozoa, together with an improvement of sperm motility in these samples. Therefore, the authors speculated that changes in the seminal plasma composition may influence spermatozoa motility and kinematic parameters [45].

## 5. Limitations

The results of this study have been obtained in a single center. To corroborate these findings, larger trials should be designed with prospective and randomized protocols. Moreover, a limitation of our study is that the selection procedure may have influenced the possible beneficial effect of shorter abstinence. A more suitable experimental model could be to evaluate ART outcome (fertilization rate, embryo quality, pregnancy rate, and live birth rate) using spermatozoa from the first and second ejaculate by splitting the oocytes when they are retrieved in an adequate number. 

Furthermore, another limit of our study is that, while full sexual intercourse has been consistently found able to increase the circulating levels of testosterone in patients recovering from erectile dysfunction [46,47], we do not have, currently, direct evidence that this mechanism may play a role in the results found here. For this purpose, a dedicated protocol is currently ongoing.

## 6. Conclusions

With the widespread use of ART to treat couple infertility, improved sperm quality has become essential for better outcomes in ART cycles. In fact, by using better quality spermatozoa, it should be possible to obtain a greater number of good quality embryos. In recent years, a growing number of studies have supported beneficial effects on sperm parameters when semen was collected after a short length of abstinence. Consequently, the present study shows that a second ejaculate obtained after a very short time from the first could represent a simple and useful strategy to obtain spermatozoa with better parameters and to improve the results of ART in patients with abnormal sperm parameters and, particularly, in those with severe OA. Better ICSI results could be achieved by this simple, non-invasive, and inexpensive step. Certainly, the live birth rate is a multifactorial index and other important factors must be considered to obtain the final result, including oocyte quality and endometrial receptivity. However, the optimization of the abstinence length together with an adequate diagnostic and therapeutic workup and proper preparation of infertile couples before ART can contribute to an increase in their ART success rate. Personalization of ART procedure should be regarded as the gold standard for optimization, without leaving the solution to the severe male factor in infertility simply to the ICSI technique. Finally, further studies are needed to investigate the underlying molecular mechanisms and the role of epigenetic modifications in improving sperm quality after a very short period of abstinence.

## Figures and Tables

**Table 1:** Comparison of female characteristics between Group 1 and Group 2.

	Group 1	Group 2	*p*-Value
Age (years) ^a^	38.1 ± 4.7	37.1 ± 5.5,	0.143
Antral follicle count ^a^	11.2 ± 6.8	11.8 ± 7.4	0.68
Total FSH dose administered (IU) ^a^	2731.7 ± 1515.7	2600.4 ± 1473.9	0.48
Causes of female infertility Recurrent pregnancy loss (%) Endometriosis (%) Pelvic (%) Tubal (%) Polycystic ovarian syndrome (%)	24 29 22 16 9	24 24 12 35 6	1 0.7 0.3 0.07 0.56

^a^ Mean ± standard deviation. FSH = follicle stimulating hormone.

**Table 2:** Sperm parameters (mean ± SEM) of patients of Groups 1 (*n* = 223) and 2 (*n* = 90).

	Group 1	Group 2	*p*-Value
Concentration (mil/mL)	41.1 ± 2.8	28.8 ± 3.4	0.005 *
Total motility (%)	45.4 ± 1.6	37.2 ± 2.3	0.035 *
Progressive motility (%)	25.3 ± 1.2	18.8 ± 1.0	0.024 *
Normal morphology (%)	13.4 ± 0.8	11 ± 1.1	0.07

* *p* < 0.05.

**Table 3:** Sperm parameters (mean ± SEM) of the two ejaculates of the male partners in Group 2 couples (*n* = 90).

	First Ejaculate	Second Ejaculate	*p*-Value
Concentration (mil/mL)	28.8 ± 3.4	31.6 ± 3.9	0.07
Total motility (%)	37.2 ± 2.3	47.7 ± 2.4	<0.0001 *
Progressive motility (%)	18.8 ± 1.7	25.9 ± 1.7	<0.0001 *
Normal morphology (%)	11 ± 1.1	11.2 ± 1.1	0.054

* *p* < 0.05.

**Table 4:** ICSI outcomes of the couples of the Group 1 (*n* = 223) and 2 (*n* = 90).

	Group 1	Group 2	*p*-Value
Fertilization rate (%)	90%	92%	0.3
Implantation rate (%)	15%	20%	0.09
Clinical pregnancy rate (%)	20%	31%	0.001 *
Live birth delivery rate (%)	18%	22%	0.4
Miscarriage rate (%)	16%	15%	0.9
Birth weight (g) ^a^	3154.5 ± 510.3	3007.5 ± 466.3	<0.05 *
Type of birth Natural birth (*n*) Scheduled cesarean (*n*) Unplanned cesarean (*n*) Twin Birth (%) Multiple Birth (%)	16 24 1 29 0	12 8 0 35 0	

^a^ Mean ± standard deviation; * *p* < 0.05.
